# 1101. Implementing a Beta-Lactam Therapeutic Drug Monitoring Program: Experience from a Large Academic Medical Center

**DOI:** 10.1093/ofid/ofab466.1295

**Published:** 2021-12-04

**Authors:** Venugopalan Veena, Malva Hamza, Barbara A Santevecchi, Kathryn DeSear, Kartikeya Cherabuddi, Charles A Peloquin, Charles A Peloquin, Mohammad H Al-Shaer

**Affiliations:** 1 University of Florida, College of Pharmacy, Gainesville, FL; 2 University of Florida College of Pharmacy, Gainesville, Florida; 3 University of Florida Health Shands Hospital, Gainesville, Florida; 4 University of Florida, Gainesville, FL

## Abstract

**Background:**

Beta-lactams (BL) are the cornerstone of antimicrobial treatment for infections. Beta-lactam therapeutic drug monitoring (BL-TDM) optimizes drug concentrations to ensure maximal efficacy and minimal toxicity. The goals of this study were to describe the implementation process of a BL-TDM program and to further describe our experience using BL-TDM in clinical practice.

**Methods:**

This was a retrospective review of adult patients with available BL-TDM between January 2016 and November 2019 at the University of Florida (UF) Health Shands Hospital. Total serum concentrations of BL were measured in the Infectious Diseases Pharmacokinetics Lab (IDPL) at UF, using a validated ultrahigh pressure liquid chromatography assay with triple quadrupole mass spectroscopy (LC-MS-MS). At our institution, TDM is available for 11 BLs and in-house assays are performed from Mon-Fri for most BLs.

**Results:**

A total of 3,030 BL concentrations were obtained. An analysis was performed on the first BL-TDM encounter in 1,438 patients. The median age was 57 years (IQR, 41-69) and the median BMI was 27.5 kg/m^2^ (IQR, 22.5-34.5). On the day of BL-TDM, the median serum creatinine was 0.83 (IQR, 0.59-1.30). Fifty-one percent of patients (n=735) were in an ICU at the time of BL-TDM with a median SOFA score of 6 (IQR, 3-9). BL-TDM was most frequently performed on cefepime (61%, n=882), piperacillin (15%, n=218), and meropenem (11%, n=151). The BL was administered as a continuous infusion in 211 (15%) patients. An interim analysis of 548 patients showed that BL-TDM was performed a median of 2 days (IQR, 1-4) from the start of BL therapy and resulted in a dosage adjustment in 26% (n=145).

**Conclusion:**

BL-TDM was performed in older, non-obese patients with normal renal function. Over half of the evaluated patients were in an ICU at the time of TDM. This finding emphasizes the value of BL-TDM in the ICU setting because altered pharmacokinetics during critical illness has been linked to enhanced BL clearance. Interestingly, BL-TDM resulted in dosage adjustment in 1 in 4 patients who were receiving licensed BL dosing regimens, thus highlighting the role of TDM in dose individualization. BL-TDM was performed most commonly within the 72-hours of therapy initiation. Early BL-TDM has been shown to improve patient outcomes and should be promoted.

**Disclosures:**

**Venugopalan Veena, PharmD**, **Melinta** (Other Financial or Material Support, Received a stipend for participation in a drug registry)**Merck** (Other Financial or Material Support, Received a stipend for participation in a drug registry) **Charles A. Peloquin, Pharm.D.**, Nothing to disclose

